# Patient perspectives on the use of artificial intelligence in prostate cancer diagnosis on MRI

**DOI:** 10.1007/s00330-024-11012-y

**Published:** 2024-08-14

**Authors:** Stefan J. Fransen, T. C. Kwee, D. Rouw, C. Roest, Q. Y. van Lohuizen, F. F. J. Simonis, P. J. van Leeuwen, S. Heijmink, Y. P. Ongena, M. Haan, D. Yakar

**Affiliations:** 1 University Medical Center Groningen, Groningen, Netherlands; 2 Martini Hospital, Groningen, Netherlands; 3 Twente Technical University, Enschede, Netherlands; 4 Dutch Cancer Institute, Amsterdam, Netherlands; 5 University of Groningen, Groningen, Netherlands

**Keywords:** Patient preference, Artificial intelligence, Questionnaire, Prostate cancer, Magnetic resonance imaging

## Abstract

**Objectives:**

This study investigated patients’ acceptance of artificial intelligence (AI) for diagnosing prostate cancer (PCa) on MRI scans and the factors influencing their trust in AI diagnoses.

**Materials and methods:**

A prospective, multicenter study was conducted between January and November 2023. Patients undergoing prostate MRI were surveyed about their opinions on hypothetical AI assessment of their MRI scans. The questionnaire included nine items: four on hypothetical scenarios of combinations between AI and the radiologist, two on trust in the diagnosis, and three on accountability for misdiagnosis. Relationships between the items and independent variables were assessed using multivariate analysis.

**Results:**

A total of 212 PCa suspicious patients undergoing prostate MRI were included. The majority preferred AI involvement in their PCa diagnosis alongside a radiologist, with 91% agreeing with AI as the primary reader and 79% as the secondary reader. If AI has a high certainty diagnosis, 15% of the respondents would accept it as the sole decision-maker. Autonomous AI outperforming radiologists would be accepted by 52%. Higher educated persons tended to accept AI when it would outperform radiologists (*p* < 0.05). The respondents indicated that the hospital (76%), radiologist (70%), and program developer (55%) should be held accountable for misdiagnosis.

**Conclusions:**

Patients favor AI involvement alongside radiologists in PCa diagnosis. Trust in AI diagnosis depends on the patient’s education level and the AI performance, with autonomous AI acceptance by a small majority on the condition that AI outperforms a radiologist. Respondents held the hospital, radiologist, and program developers accountable for misdiagnosis in descending order of accountability.

**Clinical relevance statement:**

Patients show a high level of acceptance for AI-assisted prostate cancer diagnosis on MRI, either alongside radiologists or fully autonomous, particularly if it demonstrates superior performance to radiologists alone.

**Key Points:**

- *Prostate cancer suspicious patients may accept autonomous AI based on performance*.
- *Patients prefer AI involvement alongside a radiologist in diagnosing prostate cancer*.
- *Patients indicate accountability for AI should be shared among multiple stakeholders*.

## Introduction

Artificial intelligence (AI) models for detecting significant prostate cancer (PCa) on magnetic resonance imaging (MRI) scans have reached performance levels comparable to expert radiologists, showcasing their potential for integration into clinical settings [1–4]. However, successful clinical integration of these AI systems requires more than just diagnostic accuracy; trust in AI diagnosis and accountability for misdiagnosis have been appointed as key challenges for the future of the field [5, 6].

Trust in AI diagnosis of PCa suspicious patients is understudied. Although there are existing studies on how patients and radiologists perceive AI in radiology, those findings might not directly apply to PCa patients [7–9]. Patient perspectives on AI implementation can differ based on settings and specific applications [8], warranting an evaluation of the unique PCa patient population that generally consists of older males. In prostate MRI assessment, the standard of care is diagnostic assessment by a single radiologist. The current landscape of AI models presents a variation in potential support for the radiologist. Support can range from models designed to have a second look to those striving for full autonomy [10, 11]. The understudied patient responses to these innovations and their acceptance of different levels of automation have become increasingly relevant. Therefore, weaving patient perspectives into the development and deployment narrative is essential. This ensures that the AI tools developed meet the technical standards and resonate with and garner trust from the patients they are designed to serve.

The future implementation of AI in PCa detection also raises questions regarding responsibility for misdiagnosis [12]. The utilization of AI for PCa detection on MRI deviates from the standard of care, placing medical doctors in a position of responsibility for any mistakes the AI makes [5]. This current responsibility of doctors when deviating from the standard of care may hinder the implementation of novel AI systems. Therefore, investigating the patients’ views on responsibility is paramount, as it indicates who could be held accountable when an AI system delivers an incorrect diagnosis.

This study investigates patients’ acceptance of AI for diagnosing PCa on MRI scans and the factors influencing their trust in AI diagnoses. In addition, it examines patients’ views on accountability for misdiagnosis by AI.

## Materials and methods

### Subjects

This prospective multicenter study was performed between January and October 2023 at three Western European medical institutes. Local institutional review board approval was obtained at each institute: institute A (METc 2022/437), institute B (MEC 2022-132), and institute C (IRBd22-233). Patients who underwent prostate MRI scans for PCa diagnosis or staging were eligible for this study. The study included patients who gave written informed consent, were 18 years or older, and completed the entire questionnaire.

### Questionnaire design

Participants received a questionnaire on paper to ask their opinion on hypothetical AI assessment of their MRI scans. The questionnaire was designed based on previously published articles, with careful consideration of the time required to complete the questionnaire [8, 13, 14]. All questions were retained in the final analysis. The questionnaire is included in Supplementary materials A. In institutes B and C, a radiology technologist handed out the questionnaire prior to the scan. In institute A, the patients received the questionnaire along with their MRI invitation letter, and the radiology technologist collected the questionnaires before the scan. The questionnaires were completed before the MRI scan to best simulate the hypothetic scenario of choosing between an AI model or a radiologist diagnosis. The questions were preceded by general information that described the scenario where AI systems and radiologists perform similarly. Demographic questions at the start of the questionnaire included the participants’ birthdate, education level, MRI experience, and cancer history. The participants’ educational level was determined based on their self-reported highest degree, in accordance with Ongena et al [14]. For cancer history, active surveillance PCa and other types of cancer were surveyed. Next, nine AI diagnosis-related questions were asked to determine the opinion on cooperation between AI and radiologists, trust in AI, and accountability for mistakes AI may make. These questions outlined various scenarios regarding autonomous reading, ranging from secondary interpretation to autonomous decision-making. The responses were measured with a 5-point Likert-type agree-disagree scale to increase the data quality, in line with recommendations by Höhne et al [15]. In the last open-ended question, patients were given the opportunity to provide additional comments.

### Analysis

Patient responses were compared to evaluate agreement with a statement. Furthermore, relative odds ratios were computed for each question in relation to demographic variables: education (per level increase), history of previous MRI scans, cancer history, and age (per year increase). If the responses met the requirements of proportional odds, an ordinal logistic regression was executed [16]. Statistical significance was determined at a threshold of *p* < 0.05, and the analysis was conducted using Rstudio, version 4.2.2, and the MASS and Brant libraries.

## Results

### Subjects

**Fig. 1:**
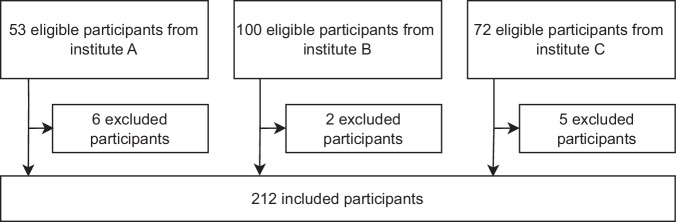
STARD diagram. Adult men who underwent prostate MRI scans between January and November 2023 at institute A, institute B, and institute C were eligible for this study. Patients with an incomplete questionnaire or multiple answers to a single question were excluded

A total of 212 patients were included in this multicenter study, with 13 patients (6%) excluded because of an incomplete questionnaire (Fig. 1). Table 1 provides an overview of the patient characteristics. The patient’s ages ranged between 31 and 83 years (median = 69 years, IQR = 10 years). The majority of patients had college-level education (54%, *n* = 114), followed by vocational education (31%, *n* = 66), and finally, high school education (15%, *n* = 32). Additional comments were given by 33 patients (16%); see Supplementary materials B.

**Table 1:** Patient characteristics

Variable		*n* (%)
Age (years)	Below 50	7 (3%)
	Between 50 and 75	153 (72%)
	Older than 75	52 (25%)
Previous experience with MRI	No	136 (64%)
	Yes	76 (36%)
History of prostate cancer	No	176 (83%)
	Yes	36 (17%)
History of cancer (other than prostate cancer)	No	42 (20%)
	Yes	170 (80%)
Education	High school	144 (54%)
	Vocational	66 (31%)
	College	32 (15%)

### Patients’ responses

**Fig. 2:**
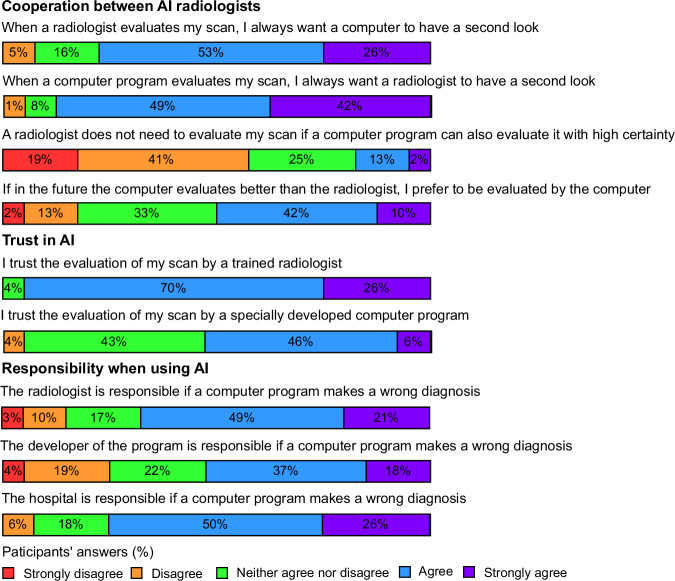
Patients’ responses to questions about their views on AI assessment of their prostate MRI scan

The responses by patients on the survey are shown in Fig. 2. Most patients preferred AI involvement alongside a radiologist in diagnosing PCa: 79% of the participants would like a second opinion by a computer program after a radiologist’s diagnosis, and 91% want a radiologist to have a second look after a computer diagnosis. AI with a high certain diagnosis was accepted as the sole decision-maker by 15% of the respondents. AI outperforming radiologists would be accepted by 52%. Interestingly, a large proportion of patients (43%) was indecisive about trusting in a computer program evaluating their scans. Respondents had a higher trust in the evaluation by a radiologist compared to a computer program (96% trust a radiologist, 52% trust a computer program). The respondents indicated that the hospital (76%), radiologist (70%), and program developer (55%) should be held accountable for misdiagnosis. The additional comments most often showed a doubtful attribute toward AI involvement in PCa diagnosis (*n* = 8, 24%), followed by a positive attribute (*n* = 7, 21%), and a resistant attribute (*n* = 3, 9%). In terms of the responsibility of AI involvement in PCa diagnosis, the additional comments expressed a preferred radiologists’ responsibility (*n* = 4, 12%), followed by shared responsibility between radiologist, program developer, and hospital (*n* = 2, 6%), and hospital responsibility (*n* = 1, 3%). All comments can be found in Supplementary materials B.

### Logistic regression

In all statements, the proportional odds assumption was satisfied, and ordinal logistic regression was performed. Table 2 shows the odd ratios to agree with a statement having higher education, MRI history, PCa history, cancer history, or higher age. Persons with higher education tended to accept AI if it would outperform radiologists and found the radiologist responsible for the failure of the computer (*p* < 0.05). Significant results were found between statements and higher age (*p* < 0.01), but the odd ratios were around 1.00, indicating no strong increase or decrease in acceptance of the statement.

**Table 2:** Relative odds ratios for each question in relation to demographic variables

Statement	Education	MRI history	PCa history	Cancer history	Age
Second opinion by a computer after radiologist evaluation	1.10 (0.91–1.35)	1.04 (0.55–1.95)	1.13 (0.51–2.48)	0.95 (0.50–1.82)	**1.01**^**†**^ **(1.01–1.01)**
Second opinion by a radiologist after computer evaluation	1.03 (0.84–1.27)	0.82 (0.44–1.55)	1.09 (0.48–2.47)	0.80 (0.41–1.56)	**1.00**^**†**^ **(1.00–1.00)**
No radiologist, when a computer program is highly certain	1.06 (0.88–1.30)	1.06 (0.57–1.98)	0.75 (0.34–1.64)	1.23 (0.66–2.30)	**1.00**^**†**^ **(1.00–1.00)**
Computer preference when outperforming a radiologist	1.26* (1.03–1.53)	1.60 (0.85–2.96)	1.33 (0.61–2.90)	1.61 (0.85–3.06)	**1.00**^**†**^ **(1.00–1.00)**
Trust in the evaluation of a computer program	0.86 (0.69–1.06)	1.05 (0.55–2.00)	0.93 (0.40–2.17)	0.97 (0.50–1.86)	**0.99**^**†**^ **(0.99–0.99)**
Trust in the evaluation of a radiologist	1.19 (0.92–1.52)	1.26 (0.62–2.56)	0.95 (0.38–2.36)	1.12 (0.54–2.34)	**1.01**^**†**^ **(1.01–1.01)**
Radiologist’s accountability for computer program misdiagnosis	1.26* (1.03–1.53)	0.73 (0.40–1.34)	0.97 (0.45–2.09)	0.65 (0.34–1.22)	1.00 (1.00–1.00)
Program developer’s accountability for computer program misdiagnosis	0.84 (0.69–1.02)	1.26 (0.68–2.33)	0.55 (0.25–1.20)	0.66 (0.35–1.24)	**0.98**^**†**^ **(0.98–0.98)**
Hospital’s accountability for computer program misdiagnosis	1.14 (0.94–1.38)	0.69 (0.37–1.29)	1.15 (0.52–2.55)	0.75 (0.38–1.45)	**1.00**^**†**^ **(1.00–1.00)**

The odds ratios with 95% confidence intervals were calculated with ordinal logistic regression

*AI* artificial intelligence, *ref* reference

The bold numbers represent statistically significant results

* Indicates *p* < 0.05

^†^ Indicates *p* < 0.01

## Discussion

This study delved into patients’ perspectives on using AI for MRI-based PCa diagnoses and the factors shaping their trust in such AI-involved diagnoses. Results indicated a pronounced preference for AI to aid a radiologist’s judgment, with 79% of patients supporting a computer-generated second opinion after a radiologist’s initial diagnosis, and 91% endorsing an AI’s primary opinion subject to a radiologist’s oversight. Despite this, only a minority of 15% favored a standalone highly certain AI diagnosis without a radiologist’s input. Nevertheless, most patients (52%) were open to fully autonomous AI, provided it surpassed a radiologist in diagnostic accuracy. The study further emphasized that in instances of misdiagnosis, participants deemed the hospital, radiologist, and AI program developer accountable, in descending order of accountability.

Overall, our results underscore that a vast majority of patients undergoing prostate MRI are receptive to AI involvement (79–91%), either as a secondary or primary evaluator. This aligns with current AI capabilities and available commercial products. Some studies and products utilize AI to read prostate MRIs initially and propose findings for radiologist approval, giving radiologists discretion to accept or discard results [17]. While there is general agreement on some level of AI involvement, the acceptance drops to 15% for standalone AI systems with high certainty, absent radiologist involvement. However, if AI were to outperform radiologists, the majority would likely accept it, suggesting a potential shift toward increased automation by AI. Although many radiology algorithms are not yet ready for this advanced level, ongoing research suggests it is feasible for specific algorithms [11]. Educating patients about the benefits of the current healthcare system (e.g., increased diagnostic performance and productivity gain) might further persuade them toward this approach. Notably, 33% of the participants neither agree nor disagree with automated AI reading when the AI outperforms radiologists, indicating a potential target for additional education and information about AI technology.

This study also investigated patients’ views on accountability in AI system performance. The hospital, radiologist, and AI program developer were identified as responsible parties. Currently, medical doctors are responsible for patient care outcomes, facing liability for deviations from standard protocols [5]. As standard care increasingly integrates medical AI, this accountability landscape may shift, with AI developers playing a more influential role in patient outcomes and facing greater legal responsibility [5, 6, 18]. However, each setting and workflow in healthcare has unique specifics that influence how existing laws must be adapted. Future collaborative efforts among hospitals, radiologists, AI developers, and legal experts are essential to tailor these adjustments appropriately.

While existing literature provides insights into patients’ perspectives on AI, its applicability to patients undergoing a prostate MRI might be limited. This limitation arises due to potential variations in views influenced by diverse settings and applications, as highlighted in previous studies [8, 9]. Our study was specifically designed to address this gap by focusing solely on patients undergoing prostate MRI for the diagnosis or staging of prostate cancer (PCa). The questionnaire was administered prior to the MRI to replicate the clinical environment and account for the anxiety associated with awaiting a diagnosis. Although another study has investigated patient trust in AI among patients undergoing prostate MRI, direct comparisons are challenging because their study encompassed all diagnostic and therapeutic interventions for PCa, with 58% of patients visiting for radical prostatectomy [19]. Their findings indicated a slightly reduced preference for AI involvement in the diagnosis (67% AI-assisted diagnosis and 1% AI alone diagnosis), without a correlation between education and trust in AI. Besides a different patient cohort, the differences might be attributed to the double focus of their questionnaire on diagnosis and communication capabilities of AI: patients do not prefer AI communication but do prefer AI involvement in the diagnosis [19].

The role of radiologists in patients’ trust in AI-assisted PCa diagnosis was also highlighted in a focus group study [20]. Their results showed that participants’ trust depended not only on AI technology but also on the radiologists, whom they trusted to utilize thoroughly tested, beneficial tools. In addition, the preference for a human-centered approach was also expressed in terms of the importance of patient-professional relationships, including empathy in communication. While AI can aid in the detection of PCa, a human-centered approach was preferred to provide a balance between diagnostic accuracy and human intuition [20].

When comparing our study to the broader literature on AI in healthcare, our study identified relatively large support for a standalone AI system, particularly if it demonstrated superior performance compared to a radiologist. This contrast might be attributed to the unique perspectives of the patient cohort undergoing MRI for PCa diagnosis or staging [8]. For instance, a study conducted in the radiology department in 2021, which focused on women’s acceptance of AI in mammogram interpretation, found that 46% of participants preferred AI as a secondary reader [14]. However, our results indicate a higher acceptance rate, with 80% of participants favoring a computer program’s second opinion after a radiologist’s initial diagnosis in PCa detection. This discrepancy may suggest gender-based differences in perceptions of AI in healthcare [8]. Furthermore, the growing acceptance of AI could also be influenced by the rapid development and increasing visibility of AI algorithms, such as large language models like ChatGPT, which may have enhanced public awareness and trust in AI capabilities over time. Therefore, conducting targeted studies that directly address specific audiences and scenarios is crucial. Such studies will help to accurately delineate the real boundaries and acceptability of AI algorithms in various medical contexts [14].

This study encountered limitations, primarily due to its research focus on Western European male patients with higher education levels. As such, the generalizability of our results may be limited, given that female patients or other populations may hold different perspectives [8, 20, 21]. Additionally, this study represents a snapshot of the continuum of ongoing research. Future surveys need to evolve alongside the changing landscape of technologies, reflecting various stages of performance and automation. As a follow-up study, it would be intriguing to explore patients’ familiarity with AI, such as ChatGPT, and then categorize their AI acceptance based on their AI familiarity.

With PCa detection AI reaching expert-level performance, the vast majority of patients showed a preference for AI involvement alongside a radiologist in diagnosing PCa on MRI. Autonomous AI was accepted by a small majority on the condition that AI outperforms a radiologist. Moreover, higher education levels were linked to increased trust in AI. Respondents held the hospital, radiologist, and program developers accountable for misdiagnosis, in descending order of accountability.

## Supplementary information

ELECTRONIC SUPPLEMENTARY MATERIAL

## Abbreviations

AI: Artificial intelligence
MRI: Magnetic resonance imaging
PCa: Prostate cancer
