# Supplementary material for: Patient perspectives on the use of artificial intelligence in prostate cancer diagnosis on MRI
Source: Eur Radiol. 2024 Aug 14;35(2):769–75. doi: 10.1007/s00330-024-11012-y (PMC11782406; doi:10.1007/s00330-024-11012-y)
Supplement: Supplementary file 1 — ELECTRONIC SUPPLEMENTARY MATERIAL [file 330_2024_11012_MOESM1_ESM.pdf]

Patient Perspectives on the Use of Artificial Intelligence in Prostate Cancer Diagnosis on MRI

ELECTRONIC SUPPLEMENTARY MATERIAL

A. *Questionnaire*

Preceding information:

Research has shown that computer programs and radiologists are equally good at evaluating a prostate MRI scan. Both a computer program and a radiologist can indicate how certain or uncertain they are of their evaluation.

Statements:

1. When a radiologist evaluates my scan, I always want a computer program to look at it as well.

Strongly disagree	Disagree	Neither agrees nor disagree	Agree	Strongly agree
☐	☐	☐	☐	☐

2. When a computer program evaluates my scan, I always want a radiologist to look at it as well.

Strongly disagree	Disagree	Neither agree nor disagree	Agree	Strongly agree
☐	☐	☐	☐	☐

3. A radiologist does not need to evaluate my scan if a computer program can also evaluate it with high certainty.

Strongly disagree	Disagree	Neither agree nor disagree	Agree	Strongly agree
☐	☐	☐	☐	☐

8. I … the evaluation of my scan by a specially developed computer program.

4. If, in the future, the computer evaluates better than the radiologist, I would prefer to be evaluated by the computer.

Strongly disagree	Disagree	Neither agree nor disagree	Agree	Strongly agree
☐	☐	☐	☐	☐

5. The radiologist is responsible if a computer program makes a wrong diagnosis.

Strongly disagree	Disagree	Neither agree nor disagree	Agree	Strongly agree
☐	☐	☐	☐	☐

6. The developer of the program is responsible if a computer program makes a wrong diagnosis.

Strongly disagree	Disagree	Neither agree nor disagree	Agree	Strongly agree
☐	☐	☐	☐	☐

7. The hospital is responsible if a computer program makes a wrong diagnosis.

Strongly disagree	Disagree	Neither agree nor disagree	Agree	Strongly agree
☐	☐	☐	☐	☐

Completely distrust	Disrust	Neither trust nor distrust	Trust	Completely trust
☐	☐	☐	☐	☐

9. I … the evaluation of my scan by a trained radiologist.

Completely distrust	Disrust	Neither trust nor distrust	Trust	Completely trust
☐	☐	☐	☐	☐

Additional comments

Additional comments can be placed here:

B. ***Additional comments analysis***

Additional comments from participating patients (n=33, 16%) were translated on www.deepl.com and are provided below. In terms of attributes toward AI involvement in PCa diagnosis, the additional comments most often showed a doubtful attribute (n=8, 24%), followed by a positive attribute (n=7, 21%), and a resistant attribute (n=3, 9%). In terms of the responsibility of AI involvement in PCa diagnosis, the additional comments expressed a preferred radiologists' responsibility (n=4, 12%), followed by shared responsibility between radiologist, program developer, and hospital (n=2, 6%), and hospital responsibility (n=1, 3%).

Index	Comment	AI diagnosis	Responsibility
1	I may be old-fashioned, but I (still) have more faith in a human (radiologist?) than a machine (computer?).	Resistant	-
2	I notice that you are looking for innovations! Very good!	-	-
3	There will come a time, when everything goes through AI!	-	-
4	As long as diagnosis is in its infancy, monitoring by the radiologist is a requirement. Perhaps that can change in the future!	Doubtful	-
5	Shared responsibility.	-	Shared
6	In my opinion, the radiologist is always responsible for the diagnosis. After all, the	-	Radiologists

	latter also determines to what extent the computer program's diagnosis can be trusted.		
7	Given the introduction of new techniques, that includes acceptable risks. So responsibility yes, liability no.	-	-
8	Nice questionnaire, especially for someone working in ict.	-	-
9	In time, a computer program, AI-driven or otherwise, can more easily perceive what might elude the human eye. In addition, such a program can also draw completely nonsensical conclusions based on incorrect assumptions that even the programmer in question can only figure out by thoroughly inspecting the code and thus retracing the erroneous conclusions. Human inspection by a radiologist remains necessary.	Doubtful	-
10	Anyone can make mistakes, including a radiologist.	-	-
11	Most confidence I would have in an initial assessment by the computer program with a “second” opinion by the radiologist. Nice development.	Positive	-
12	Where can I possibly obtain more information about this computer program?	-	-
13	Depends on computer experience. Definitely recommended as a supplement.	Positive	-
14	The degree of confidence is obviously influenced by the strength with which the initial proposition is substantiated	Doubtful	-
15	Both are perfectly capable of assessing a scan. However, hospital, radiologist and software developer remain responsible for claims.	Positive	Shared
16	Certify programs.	-	-
17	See now scan computer program as supplement (kind of second opinion).	Positive	-
18	It is difficult to give the right answers now when insight into the software to be developed is not clear or possible.	Doubtful	-
19	Questions of responsibility are very specific and therefore unanswerable.	-	-
20	Complicated ... not ultimately responsible. That can only be one party (the radiologist), but co-responsible? If I get good evidence.	-	Radiologists
21	The radiologist remains responsible. Everybody is different. Can this be standardized?	-	Radiologists

22	Staff out? Computer program in!!! Where is the human dimension?	Resistant	-
23	Question 6: “better” is differentiating too vaguely. Perhaps using >%<? 7,8 and 9 I answered “agree” in all cases, to consciously maximize the human factor. Humans remain responsible, but maximum technical support is desirable.	Positive	Radiologists
24	There is a difference between early versions of the program and later improved versions.	-	-
25	At this time that the computer with the software is an additional tool of the radiologist and therefore not a replacement!	Positive	-
26	Please keep a human being there at all times.	Doubtful	-
27	Important that computers and people are checked. One hundred percent certainty can almost never be guaranteed.	Doubtful	-
28	Always double check.	Doubtful	-
29	As could be seen from the answers, I would like to await developments in computers.	Resistant	-
30	The computer serves the human (for now). The developer serves the physician. The physician serves the human. The hospital facilitates the physician and is ultimately responsible.	-	Hospital
31	I have great faith in technology , but a check is always better and responsibility should just be looked at legally.	Positive	-
32	There is still much to explore.	Doubtful	-
33	I assume the program has been extensively tested and is good.	-	-

Table 1. This table shows all additional comments made by participants of the study. In addition, it shows the AI diagnosis classification (resistant, doubtful, or positive) and the responsibility classification (radiologists, program developer, hospital, or shared).
